# Insight into the genomic diversity and relationship of *Astragalus glycyphyllos* symbionts by RAPD, ERIC-PCR, and AFLP fingerprinting

**DOI:** 10.1007/s13353-015-0285-6

**Published:** 2015-05-01

**Authors:** Sebastian Gnat, Wanda Małek, Ewa Oleńska, Aleksandra Trościańczyk, Sylwia Wdowiak-Wróbel, Michał Kalita, Magdalena Wójcik

**Affiliations:** Department of Veterinary Microbiology, University of Life Sciences, 12 Akademicka st., 20-950 Lublin, Poland; Department of Genetics and Microbiology, University of Maria Curie-Skłodowska, 19 Akademicka st., 20-033 Lublin, Poland; Department of Genetics and Evolution, Institute of Biology, University of Białystok, 20B Świerkowa st., 15-950 Białystok, Poland

**Keywords:** *Astragalus glycyphyllos* symbionts, DNA fingerprinting, Genome polymorphism, Genomic relationship, Rhizobia

## Abstract

We assessed the genomic diversity and genomic relationship of 28 *Astragalus glycyphyllos* symbionts by three methodologies based on PCR reaction, i.e., RAPD, ERIC-PCR, and AFLP. The AFLP method with one *Pst*I restriction enzyme and selective PstI-GC primer pair had a comparable discriminatory power as ERIC-PCR oneand these fingerprinting techniques distinguished among the studied 28 *A. glycyphyllos* symbionts 18 and 17 genomotypes, respectively. RAPD method was less discriminatory in the genomotyping of rhizobia analyzed and it efficiently resolved nine genomotypes. The cluster analysis of RAPD, ERIC-PCR, and AFLP profiles resulted in a generally similar grouping of the test strains on generated dendrograms supporting a great potential of these DNA fingerprinting techniques for study of genomic polymorphism and evolutionary relationship of *A. glycyphyllos* nodulators. The RAPD, ERIC-PCR, and AFLP pattern similarity coefficients between *A. glycyphyllos* symbionts studied was in the ranges 8–100, 18–100, and 23-100 %, respectively.

The root and stem nodulating bacteria, collectively known as rhizobia, form symbiotic interactions with leguminous plants to convert atmospheric nitrogen into ammonia for micro- and macrosymbiont uptake. Rhizobium-legume symbiosis is agriculturally and ecologically important for many purposes including the production of food, fodder, enrichment of the soil in nitrogen, thereby the application of nitrogenous fertilizers is reduced (Perret et al. 2000). In many countries the legumes are routinely inoculated with rhizobia which are highly competitive to nodulate host in the presence of native soil rhizobia (Ravikumar 2012). To distinguish legume inoculant rhizobia from a native population occurring in soil, reliable methodsfor bacteria identification and differentiation are needed.

In recent years, many molecular techniques have been developed for typing and assessing genomic diversity of bacteria. Among the best known methods exploiting PCR are: random amplified polymorphic DNA (RAPD) (Harrison et al. 1992), repetitive element-based polymerase chain reaction (ERIC- and REP-PCR) (Versalovic et al. 1991), and amplified fragment length polymorphism (AFLP) (Blears et al. 1998). All of them generate markers that are specific for a given genome and require no prior genome sequence knowledge.

In this study, rhizobia isolated from root nodules of leguminous plant *A. glycyphyllos* growing in Poland were characterized for genomic diversity and relationship by three commonly used DNA fingerprinting techniques based on PCR reaction namely: RAPD, ERIC-PCR, and AFLP. Total genomic DNAs from 28 *A. glycyphyllos* symbionts (AG1-AG29) were used in PCR reaction with 10-base oligonucleotide (5’-GGAAGTCGCC-3’) as a random primer containing 70 % of GC. The use of high GC% primer in RAPD method was dictated by high GC content in rhizobial genomes (Jarvis et al. 1997) and served to generate sufficiently high number of amplicons. Additionally, it was suggested that random primers anneal more strongly if they are high in GC content, particularly at 3’ terminal end from which the synthesis of the DNA amplicons is initiated (Goodfellow and O’Donnell 1993). The results obtained in this study are presented in Fig. 1. RAPD-PCR, generated fingerprints of relative complexity consisting from three to seven DNA bands per strain. Only 7 % of the bacterial strains, i.e., AG10, AG12, AG22, and AG26 possessed a unique, strain specific amplification patterns. Quantitative comparison of RAPD profiles among the rhizobia specific for *A. glycyphyllos*, according to the presence or absence of amplified DNA segments, was used to produce binary matrix and to construct a dendrogram presenting genomic polymorphism and relationship of bacteria (Fig. 1). In the dendrogram derived from RAPD fingerprinting data, the rhizobia tested were split into two genomically divergent clusters (at DNA pattern similarity level of 45 %) and one independent lineage outside of all other bacteria. Eighteen *A. glycyphyllos* nodulators were grouped within one major cluster comprising three clear subgroups and one independent lineage (AG10 strain). The largest of these subgroups contained 12 bacteria with identical RAPD fingerprints. Two other ones were represented by three and two strains with the same RAPD patterns. The second main cluster comprised six nodule isolates and four of them exhibited identical DNA profiles. On the most distant branch of RAPD tree, AG26 strain and three other *A. glycyphyllos* symbionts (AG13-AG15) with 63 % of amplicons characteristic only for them were situated. The overall topology of this dendrogram is very similar to that of phylogram generated in our earlier studies on the basis of RFLP of 16S rDNA which presents the close phylogenetic relationship of *A. glycyphyllos* symbionts with the genus *Mesorhizobium* species (Gnat et al. 2014). On the 16S rDNA-RFLP phylogram, *A. glycyphyllos* nodule isolates formed three major lineages with identical strain composition as on RAPD dendrogram showing that relationship of test rhizobia established by RAPD reflects their phylogeny.

**Fig. 1:**
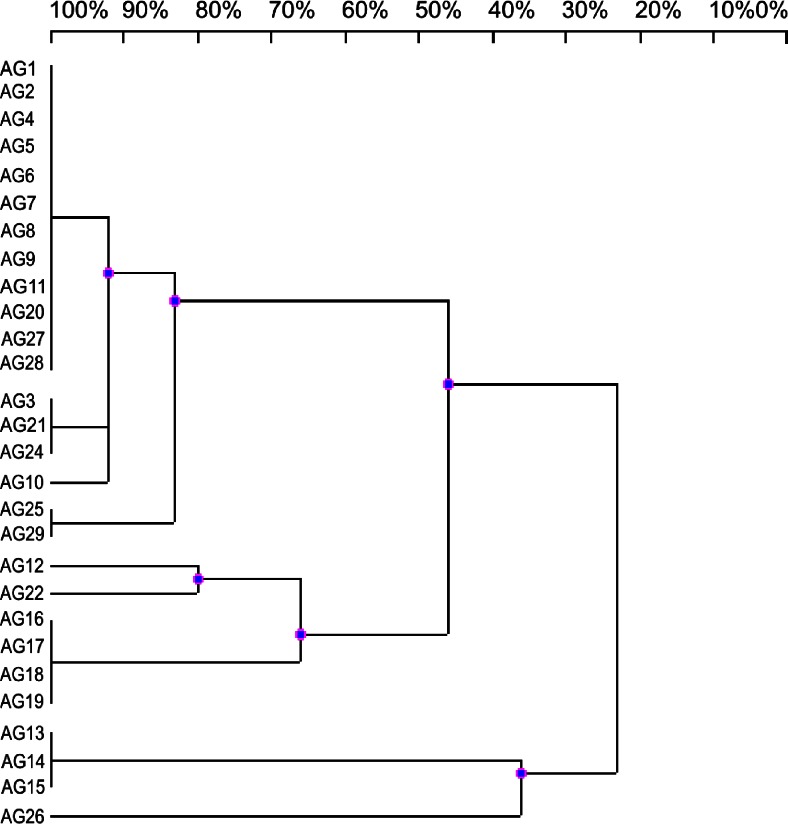
UPGMA dendrogram obtained from RAPD patterns of the *Astragalus glycyphyllos* symbionts DNA. The scale at the top of the dendrogram presents the bacterial genome similarity rate (%). AG1-AG29- *A. glycyphyllos* nodule isolates

For distinguishing bacterial strains and to study their genomic relationship, the PCR technique based on ERIC oligonucleotide primers has been very widely used (Versalovic et al. 1991). The position of ERIC-like sequences in the bacterial genomes vary among different strains and amplification products provide for them unique fingerprints when run on a agarose gel. In this study we demonstrate the presence of ERIC-like sequences in the genomes of *A. glycyphyllos* symbionts but their number was not enough to discriminate all 28 individual strains tested (Fig. 2). The amplification reaction with used ERIC primer set generated distinct polymorphic bands for reliable discrimination of 14 individual strains. The dendrogram deriving from ERIC-PCR fingerprints showed the existence of two major groups (diverging at the DNA pattern similarity level of 54 %) and one independent lineage located on the outskirt of the tree with AG13, AG14, AG15, and AG26 strains exhibiting clearly distinct DNA profiles from the remaining test rhizobia (Fig. 2). One major cluster comprised 17 *A. glycyphyllos* nodule isolates with remarkable ERIC pattern similarity among them (67-100 %), including two groups of bacterial strains with identical ERIC-PCR fingerprints. The second sister cluster comprised seven more genomically differentiated nodule isolates with ERIC-PCR profiles similarities levels from 67 to 93 %. The cluster analysis of RAPD and ERIC-PCR profiles resulted in a generally similar grouping of the test strains (Figs. 1 and 2) with four strains (AG13-AG15 and AG26) clearly diverging from the rest of *A. glycyphyllos* symbionts analyzed. The complexity of ERIC-PCR patterns was greater than that obtained with RAPD primer and enabled clear distinction of 17 genomotypes among 28 studied rhizobia whereas RAPD fingerprinting method allowed to delineate nine genomotypes. It is also worth underlining that *A. glycyphyllos* nodulators showing similar ERIC-PCR patterns and forming common clusters on ERIC dendrogram also occurred in the same clusters (except for AG24 strain) on 16S rDNA-RFLP phylogram close to the genus *Mesorhizobium* species (Gnat et al. 2014). Taking into account that 16S rDNA PCR-RFLP is recommended for tracing evolutionary history of large number of bacteria (Rossello-Mora and Amann 2001), we state that ERIC-PCR method is valuable not only for the evaluation of genomic diversity of *A. glycyphyllos* symbionts but also for the analysis of their evolutionary relationship.

**Fig. 2:**
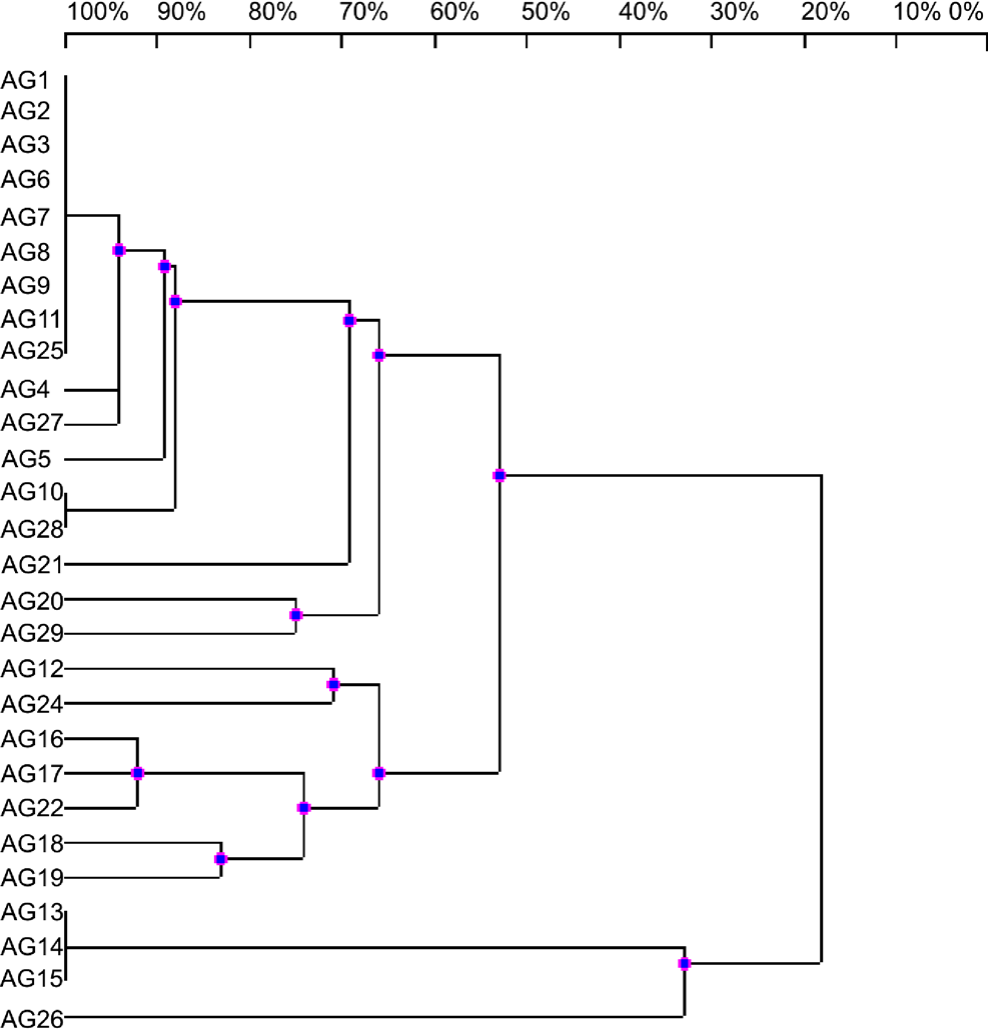
UPGMA dendrogram showing the genomic diversity of *Astragalus glycyphyllos* symbionts based on ERIC patterns. The scale at the top of the dendrogram presents the bacterial genome similarity rate (%). AG1-AG29- *A. glycyphyllos* nodule isolates

For molecular typing and assessment of relationship between *A. glycyphyllos* nodule isolates, a highly reproducible AFLP method was also used. This genome fingerprinting technique is based on the nucleotide changes within restriction sites and adjacent to them bases used for primer annealing (Blears et al. 1998; Kalita and Małek 2006). To generate an informative number of polymorphic bands in AFLP analysis the choice of suitable restriction enzyme is required. The endonuclease used should be adapted to the GC content in DNA of test bacteria. In the present study, for *A. glycyphyllos* nodule isolates profiling, the restriction enzyme *Pst*I recognizing GC rich consensus sequence 5’-CTGGAG-3’ was used based on the fact that rhizobium genome is reached in GC bases (59-66 % mol%) (Jarvis et al. 1997). The efficiency of AFLP method also depends on the kind and number of selective nucleotides at 3’ end of the restriction enzyme primers (Blears et al. 1998). The primer pair PstI-GC, which contain at 3’ end GC as arbitrary nucleotides and amplify only a part of tagged restriction DNA fragments, was used in PCR reaction across 28 *A. glycyphyllos* rhizobia (Fig. 3). Among 28 *A. glycyphyllos* nodulators studied 13 strains exhibited DNA fingerprints peculiar only for them in PCR reaction. Genomic relatedness of *A. glycyphyllos* symbionts studied is visualized in the dendrogram presented in Fig. 3. In the tree based on DNA banding profiles from PCR reaction with PstI-GC primer set, strains were distributed into two major clusters at DNA profile similarity coefficient of 19 % and one independent branch comprising AG22 and AG26 strains on the outskirt of the tree (with AFLP pattern similarity coefficient to the remaining bacteria of 8 %). Eighteen nodule isolates were grouped within one cluster and ~70 % of them shared identical DNA bands. In the second major cluster, eight rhizobial strains were placed with two (AG28 + AG29) exhibiting identical DNA banding patterns.

**Fig. 3:**
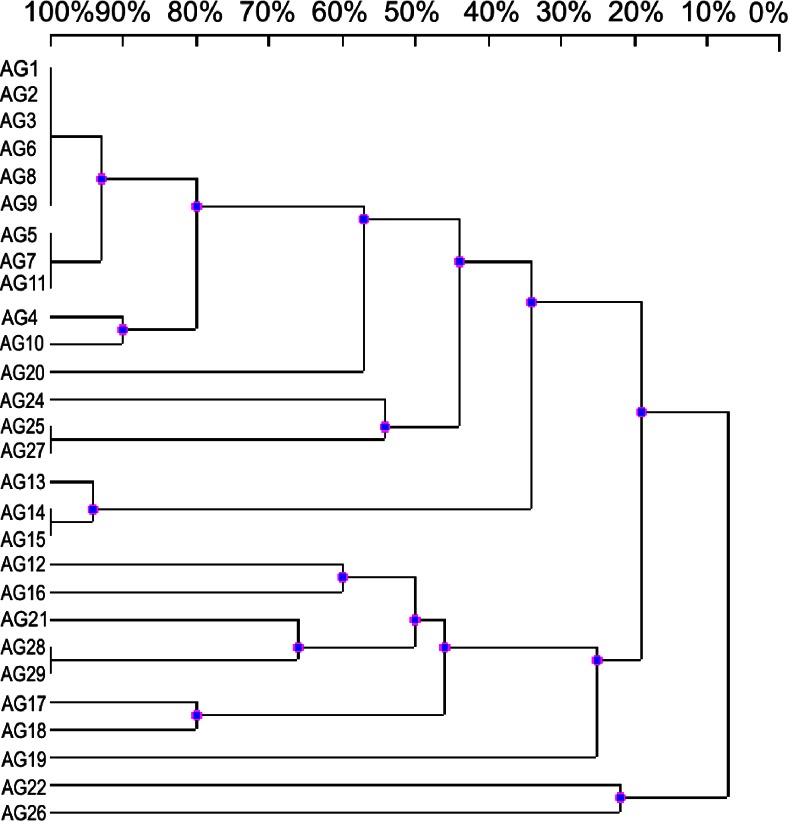
UPGMA dendrogram showing the genomic diversity of *Astragalus glycyphyllos* symbionts based on AFLP fingerprints with Pst-GC primer pair. The scale at the top of the dendrogram presents the bacterial genome similarity rate (%). AG1-AG29- *A. glycyphyllos* nodule isolates

Our results showed that simplified AFLP method with one *Pst*I restriction enzyme and selective PstI-GC primer set as well as ERIC-PCR have a comparable discriminatory power and both these fingerprinting techniques enabled differentiation of 18 and 17 genomotypes, respectively among 28 *A. glycyphyllos* symbionts studied. The grouping of *A. glycyphyllos* symbionts on AFLP dendrogram was in general concert with clustering of these rhizobia on RAPD, ERIC-PCR, and 16S rDNA-RFLP dendrograms (except for AG21, AG22, AG28, AG29 strains) (Gnat et al. 2014). All results presented in this paper supported the great potential of used PCR based fingerprinting techniques for investigating genomic polymorphism and evolutionary relationship of *A. glycyphyllos* nodulators. We suppose also, that *A. glycyphyllos* symbionts representing three main lineages on generated dendrograms may belong to three different species in the genus *Mesorhizobium*.

Additionally, we state that all these genome profiling techniques offer a convenient way to choose the right representative strains, from each genomic cluster, for further taxonomic studies such as multilocus sequence analysis (MLSA), DNA-DNA hybridization which (because of high costs) are usually carried out with only a few strains. Modern polyphasic bacterial taxonomy is based on the integration of different kinds of data, i.e., phenotypic, genomic, and phylogenetic (Rossello-Mora and Amann 2001).
